# Following Spinal Cord Injury Transected Reticulospinal Tract Axons Develop New Collateral Inputs to Spinal Interneurons in Parallel with Locomotor Recovery

**DOI:** 10.1155/2017/1932875

**Published:** 2017-09-12

**Authors:** Zacnicte May, Keith K. Fenrich, Julia Dahlby, Nicholas J. Batty, Abel Torres-Espín, Karim Fouad

**Affiliations:** ^1^Neuroscience and Mental Health Institute, University of Alberta, Edmonton, AB, Canada T6G 2E1; ^2^Department of Physical Therapy, Faculty of Rehabilitation Medicine, University of Alberta, Edmonton, AB, Canada T6G 2G4

## Abstract

The reticulospinal tract (RtST) descends from the reticular formation and terminates in the spinal cord. The RtST drives the initiation of locomotion and postural control. RtST axons form new contacts with propriospinal interneurons (PrINs) after incomplete spinal cord injury (SCI); however, it is unclear if injured or uninjured axons make these connections. We completely transected all traced RtST axons in rats using a staggered model, where a hemisection SCI at vertebra T10 is followed by a contralateral hemisection at vertebra T7. In one group of the animals, the T7 SCI was performed 2 weeks after the T10 SCI (delayed; dSTAG), and in another group, the T10 and T7 SCIs were concomitant (cSTAG). dSTAG animals had significantly more RtST-PrIN contacts in the grey matter compared to cSTAG animals (*p* < 0.05). These results were accompanied by enhanced locomotor recovery with dSTAG animals significantly outperforming cSTAG animals (BBB test; *p* < 0.05). This difference suggests that activity in neuronal networks below the first SCI may contribute to enhanced recovery, because dSTAG rats recovered locomotor ability before the second hemisection. In conclusion, our findings support the hypothesis that the injured RtST forms new connections and is a key player in the recovery of locomotion post-SCI.

## 1. Introduction

Regrowth of adult central nervous system (CNS) axons after spinal cord injury (SCI) is limited due to intrinsic factors [1] as well as by a growth inhibitory environment including myelin-associated inhibitors [2] and chondroitin sulfate proteoglycans (CSPGs) in the scar and perineuronal net [3, 4]. Still, adaptive changes in the brain and spinal cord (i.e., plasticity) do occur and are important mechanisms and treatment targets for functional recovery after CNS injuries in humans and animal models. Plasticity in the spinal cord includes changes such as axon collateral sprouting (i.e., axon growth from new or existing collaterals) [5–7], synaptic rearrangements [8–10], and changes in cellular properties [11–13].

The corticospinal tract (CST) is a descending system that has received special attention in regard to neuroplasticity after SCI [6, 8, 14, 15]. Possible reasons for this attention includes the ease of neuroanatomical tracing of cortical neurons, the simplicity of transecting the dorsally located tract in rodents, and the importance of the CST in voluntary motor control in primates [16] and humans [17]. Injured CST axons can sprout rostral to a SCI, contributing to the recovery of motor function [6, 8]. Collateral sprouting rostral to a lesion may support recovery by allowing descending CST axons to form new synaptic connections with propriospinal interneurons (PrINs). Many PrINs are commissural interneurons (i.e., crossing the midline) with many of them projecting to the lumbar enlargement; thus, providing a detour connection for the CST [7, 8]. The significance of CST axon relays via PrINs has been demonstrated in rodents with staggered SCIs [10]. In this SCI model, the animal receives two lateral hemisections at different thoracic segments on opposite sides of the spinal cord. This model is designed to completely transect all axons connecting locomotor circuits in the lumbar spinal cord with the brain and brainstem but leaves a bridge of tissue containing PrINs between the two hemisection SCIs. Due to rewiring within this bridge and changes in motor neuron properties, animals can recover substantial locomotor function compared to spinalized animals [12, 18].

The reticulospinal tract (RtST) is important for initiating walking in cats and rodents [19, 20]. It has also been noted that RtST axons have a remarkable ability for neurite outgrowth/regeneration compared to CST axons [21–23], making it a promising target for plasticity-promoting treatments. Despite this knowledge, research on collateral sprouting/plasticity of the RtST lags behind that of the CST [24].

First demonstrations that, like the CST, the RtST can form new connections to circumvent SCI were made by Filli et al. [9]. They reported increased reticulo-propriospinal contacts after unilateral cervical hemisection in adult rats. This plasticity was accompanied by substantial locomotor improvements, implicating interactions between the lesioned RtST and PrINs as important structural relays post-SCI. Given that in the study by Filli and colleagues the RtST tract was lesioned unilaterally, it is likely that plasticity in spared RtST axons also contributed to the improvements in hindlimb function [5]. Thus, it is not possible to make a clear conclusion on the contribution of the sprouting of lesioned versus spared axons on recovery. Therefore, the aim of the current study was to clarify the role of injured RtST axons and their connection to PrINs in locomotor recovery following staggered SCIs, where no spared RtST axons are present.

## 2. Materials and Methods

### 2.1. Subjects

Experiments were conducted using adult female Lewis rats (*N* = 18; Charles River Laboratories, Wilmington, MA, USA), weighing 230–270 g. The dSTAG group was made up of *n* = 10 rats, and the cSTAG group was made up of *n* = 8 rats. One of the dSTAG animals was found to have axonal sparing (see Section 2.5.4 for details), and another did not recover from the tracing surgery. One animal from the cSTAG group had a complete anatomical SCI continuous from T7 to T10. Hence, these animals were removed from the study for a final dSTAG *n* = 8 and cSTAG *n* = 7. Rats were housed in pairs in standard home-cages and kept on a 12 : 12 light-dark cycle. Water and food were provided ad libitum. Experiments were approved by the Health Sciences Animal Care and Use Committee of the University of Alberta.

### 2.2. Surgical Procedures

All surgeries were performed similar to previous descriptions [12, 25] under isoflurane anesthesia: 5% for induction and 2–2.5% for maintenance. To prevent anesthesia-induced hypothermia, surgical procedures were conducted on a heating blanket set to 37°C. At the start of surgeries, rats were shaved and their skin was disinfected with 2% chlorhexidine digluconate (Sigma-Aldrich Canada Ltd., Oakville, ON, Canada) and ETOH. Eyes were lubricated with Tears Naturale (Alcon Canada Inc., Mississauga, ON, Canada).

**Figure 1:**
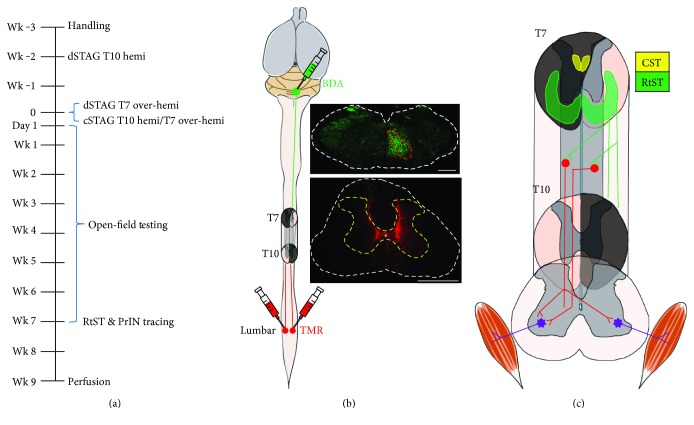
Experiment timeline and STAG SCI model. The timeline is shown in (a). The RtST was anterogradely traced with BDA, and PrINs were retrogradely traced with TMR (b). (c) shows a detailed view of the two SCIs and intact tissue bridge and outlines some of the possible reticulo-propriospinal pathways that could circumvent the SCIs to allow descending input from the RtST to lumbar motor systems. Areas of the spinal cord which are shaded dark grey correspond to the intended injuries. The CST and RtST appear in yellow and green, respectively. Traced RtST axons are shown descending in the white matter of the unlesioned hemicord at T7 (green; (c)) and stopping once reaching the second injury at T10. We hypothesize that injured RtST axons could project collaterals into the grey matter and form contacts with PrINs (red; (c)) that would then carry the signal to motor neurons (purple; (c)) below the SCIs. White dashed line, outline of cross-section. Red dashed line, gigantocellular reticular nucleus. Yellow dashed line, outline of lumbar grey matter. Scale bar = 1000 *μ*m.

The dSTAG group underwent a lateral hemisection at vertebral level T10 (right spinal cord), followed 2 weeks later by a lateral over-hemisection at vertebral level T7 (left spinal cord; Figure 1(a)). The short delay between SCIs was based on our studies where a similar delay in a staggered model resulted in functional recovery [12, 25]. For the present work, our aims were to injure all descending RtST axons and to observe some recovery in all animals (to see if this would be associated with RtST-PrIN contact formation). The staggered model with a short delay accomplished both of these goals. In contrast to the dSTAG group, the cSTAG animals received both hemisections concomitantly (received the T7 and T10 lesions in the same surgery). For the hemisection SCIs, one half of the spinal cord was cut, while for the over-hemisections, the dorsal funiculus was bilaterally transected in addition to one half of the spinal cord [10]. For the SCI surgeries, a dorsal midline skin incision was made from vertebral level T6 to T11, and the underlying muscles were dissected from the T10 and/or T7 vertebra. A dorsal laminectomy was performed on vertebra T10 and/or T7, the dura mater was lifted with fine forceps and cut open with spring scissors, and the spinal cord was transected using a custom-blade. After the hemisection(s), muscles were sutured with Vicryl 5-0 (Johnson & Johnson Medical Pty Ltd., Sydney, NSW, Australia), and the skin incision was closed with stainless steel clips (Stoelting Co., Wood Dale, IL, USA). Following each operation, the animals received 4 ml of saline (s.c.) and placed in a heated cage until fully awake. Buprenorphine (0.05 mg/kg, s.c.; Temgesic, Schering-Plough, Kirkland, QC, Canada) was given at the end of surgery and every 8 hours for 2 days to maintain postoperative analgesia. Bladder fullness was checked two to three times daily post-SCI and manually expressed as needed until the experimental endpoint.

Seven weeks post-SCI, all rats underwent surgery to inject neuronal tracers. Small cranial windows (~1 mm × 1 mm) located 0.8 mm lateral to the midline and 2.8 mm caudal of the interaural line were made using a dental drill. RtST axons were stained using the anterograde tracer biotinylated dextran amine (BDA 10000 MW; Invitrogen, Carlsbad, CA, USA). BDA (1 *μ*l of 10% BDA dissolved in 0.01 M PB) was pressure injected manually into the right gigantocellular (Gi) nucleus of the reticular formation in the brainstem using a stereotaxic frame (coordinates: 0.8 mm lateral to the midline, 2.8 mm caudal to the interaural line, and 7.6 mm deep) with a 10 *μ*l Hamilton syringe (26 s with an outer diameter of 0.47 mm and an inner diameter of 0.13 mm; Figures 1(b) and 1(c)). Next, a laminectomy of vertebra T13 was performed to expose the L3 spinal cord. PrINs located in the tissue bridge between the two hemisection SCIs and projecting to the lumbar enlargement were stained using the retrograde tracer dextran tetramethylrhodamine (TMR 10000 MW; Invitrogen). TMR (0.5 *μ*l of 10% TMR dissolved in 0.01 M PB) was pressure injected manually into the bilateral intermediate lamina of L3 (coordinates: ~200–300 *μ*m lateral to the midline, 400 *μ*m deep) using a pulled glass microelectrode mounted onto a Hamilton syringe connected to a micromanipulator (Figures 1(b) and 1(c)). Tracer injections were carried out over the span of 2 min/injection, with a 2 min waiting period before withdrawal of the syringe/microelectrode tip. Spinal tracing of PrINs with TMR was performed immediately following BDA brainstem tracing. One animal from the cSTAG group died during neuronal tracing while under surgical plane anaesthesia and was, thus, removed from the study.

### 2.3. Behavioural Testing

The BBB locomotor rating scale was used to assess functional recovery post-SCI. In short, each rat was individually assessed for 4 min by two independent observers in an open field (30 × 90 × 120 cm) and scored according to the scale developed by Basso et al. [26]. Testing was performed 1 day after bilateral SCI and continued weekly for 7 weeks. Mean group scores for the hindlimb contralateral to the T10 SCI and the hindlimb ipsilateral to the T10 SCI, as well as the average score of the two hindlimbs, were assessed.

### 2.4. Perfusion and Tissue Processing

Two weeks after tracing, the animals were euthanized with an overdose of pentobarbital (1000 mg/kg i.p.; Euthanyl; Biomeda-MTC, Cambridge, ON, Canada) and perfused with saline containing heparin followed by 4% paraformaldehyde with 5% sucrose (PFA; 0.1 M PB; pH 7.4). Spinal cords were removed, postfixed in 4% PFA overnight, and cryoprotected in 30% sucrose for 3 days. The perfused CNS was divided into blocks for cutting, including the cervical spinal cord (C1), T7 SCI, T10 SCI, the thoracic spinal cord between the two hemisection SCIs (T8-T9), and the lumbar spinal cord (L3). Individual tissue blocks were coated in OCT cryoprotectant (Sakura Finetek, Torrance, CA, USA) and frozen at −60°C in 2-methylbutane (Fisher Scientific, Ottawa, ON, Canada). Blocks of spinal cord (each ~4 mm long) containing the T10 and T7 SCIs, C1, and L3 and brainstems were cut in cross-section, while the thoracic spinal cord between the two SCIs was cut horizontally. All tissue sections were 25 *μ*m thick and cut with a cryostat (Cryostar NX70; Fisher Scientific). Sections were mounted onto slides (Fisher Scientific) and stored at −20°C.

### 2.5. Histology and Analysis

All histological analyses were performed under blinded conditions. All tissue sections were dehydrated at 37°C and rehydrated by rinsing 2 or 3 × 10 min in Tris-buffered saline (TBS). Tissue from each of the blocks was processed as follows.

#### 2.5.1. Lesion Size

Slides containing cross-sections from the T7 and T10 SCI sites were immersed in 0.5% cresyl violet for 4 min. Slides were rinsed in distilled water for 2 min then serially dehydrated in increasing ethanol concentrations: 50%, 75%, and 99% for 2 min each. This was followed by clearing 2 × 2 min in xylene. Finally, samples were coverslipped with Permount (Fisher Scientific).

Cresyl violet-stained sections were imaged under bright-field microscopy using a Leica DMLB microscope equipped with a 5x (0.15 NA) objective lens (Leica Microsystems Inc., Richmond Hill, ON, Canada). The maximal lesion extent was drawn on schematics of the cross-sections, and the lesioned area was calculated as a percent of the total area of the cross-section. Spinal tissue was considered injured if the structural integrity of the tissue was compromised, such as when cavitation occurred, the grey matter was deformed, and/or the parenchyma was infiltrated by inflammatory cells stained darkly with cresyl violet. To ensure lesion completeness, the T10 and T7 lesion schematics were overlaid and the presence of undamaged parenchyma in the following regions of interest was recorded: grey matter, dorsal horns, ventral horns, white matter, dorsolateral, and ventrolateral funiculi. In cases where tissue sparing was found, the percentage was quantified using the following formula: 100 × (area of spared tissue / total cross-section area).

#### 2.5.2. Evaluation of BDA Injection

Brainstems of all rats were processed to confirm the location of injection sites. Cross-sections were incubated with streptavidin-conjugated Alexa Fluor 488 (AF488; 1 : 100; Cedarlane, Burlington, ON, Canada) in 1% normal goat serum (NGS) and TBS with Triton-X (0.5%; TBS-TX). Sections were then rinsed 2 × 10 min in TBS-TX (0.5%) and 2 × 10 min in TBS. After washing, samples were coverslipped with Fluoromount G (Cedarlane), and the slide edges were sealed with nail polish. Sections were imaged using a Leica DM6000 B epifluorescence microscope equipped with a 5x (0.15 NA) objective lens (Leica Microsystems Inc.). Images were compared to representations of the Gi reticular nucleus in Paxinos and Watson [27] to confirm tracing accuracy. Tracing accuracy was analyzed in all animals. Criteria for a successful Gi tracing were injection site location in the Gi, unilateral tracing, and restriction of tracer spread to the Gi. The region of the brainstem with the brightest fluorescent staining was identified as the site of the tracer injection. Spread of tracer to other parts of the brainstem was determined based on the presence or absence of fluorescent tracer in these regions.

#### 2.5.3. Traced RtST Axon Counts

C1 cross-sections were stained in all animals as described in Section 2.5.2. Tile scan images of the C1 sections were captured using a Leica epifluorescence microscope with the 5x objective lens. The tile scan images were analyzed with ImageJ (v.1.43 *μ*; National Institutes of Health, Bethesda, MD, USA), and the number of BDA-traced RtST axons was counted using the cell counter plugin.

#### 2.5.4. RtST Axonal Sparing

To investigate whether traced RtST axons were spared, 3,3′-diaminobenzidine (DAB) was used to stain cross-sections caudal to the T10 SCI epicenter in all animals. Sections were permeabilized using 2 × 45 min in TBS-TX (0.5%) detergent. While slides were being permeabilized, an avidin/biotin complex (ABC) solution was made using the Vectastain Elite ABC HRP kit (peroxidase, rabbit IgG; Vector Laboratories Inc., Burlington, ON, Canada). The slides were placed in a humidifying chamber; ABC solution was applied over the tissue and allowed to incubate overnight at 4°C. The following day, the slides were washed 3 × 10 min in TBS then incubated in DAB stain solution (DAB peroxidase HRP reaction kit; Vector Laboratories Inc.) until the appearance of the dark-brown precipitate from the DAB reaction. This was followed by washing with TBS for 10 min, dehydration through increasing concentrations of alcohol (50%, 75%, and twice in 99% for 2 min each), and clearing twice in xylene (2 min). The slides were coverslipped with Permount (Fisher Scientific) and left to dry.

To search for any putatively spared traced RtST axons, approximately 20 sections 25 *μ*m apart were inspected per animal for a total distance assessed of 975 *μ*m. The slides were visualized under bright-field microscopy using a Leica DMLB microscope equipped with a 40x (0.75 NA) objective lens (Leica Microsystems Inc.), and using an attached camera, images were taken of one section below the injuries and traced axons counted using the cell counter plugin on ImageJ (v.1.43 *μ*; National Institutes of Health). Only one animal (from the dSTAG group) had spared traced RtST axons below the injuries. Therefore, this animal was removed from the study. To determine the ratio of spared traced axons to those originally traced with BDA, cross-sections of the brainstem of the animal were stained, imaged, and analyzed using the same protocols as with the spinal sections. Brainstem rather than C1 sections were stained with DAB and used to determine the ratio, because all C1 sections had already been processed for other analyses.

#### 2.5.5. Evaluation of TMR Injection

L3 cross-sections were coverslipped with Fluoromount G, and the coverslip was fixed in place with nail polish. Sections were analyzed to confirm accuracy of TMR tracer injections using a Leica DM6000 B epifluorescence microscope equipped with a 10x (0.3 NA) objective lens (Leica Microsystems Inc.). Tracing accuracy was analyzed in all animals. The criterion for successful L3 tracing was injection site location in the intermediate grey matter of the lumbar spinal cord contralateral to the T10 SCI and ipsilateral to the T10 SCI.

#### 2.5.6. Counting of RtST Collaterals, PrINs, and Contacts between Collaterals and PrINs, Quantifying Collateral Growth, and Assessment of Synaptophysin Signal

Slides containing horizontal sections of the spinal cord between the T7 and T10 SCIs were incubated in 10% NGS in TBS-TX for 1 hr. Next, the samples were incubated with AF488-streptavidin (1 : 100) and rabbit anti-synaptophysin antibody (1 : 200; Sigma-Aldrich Canada Ltd.) in 1% NGS in TBS-TX overnight at 4**°**C. The slides were rinsed 3 × 10 min in TBS then incubated with AF647 conjugated secondary antibodies (goat anti-mouse; 1 : 500; Invitrogen) in 1% NGS in TBS. To prevent overstaining, samples were bathed 2 × 10 min in TBS-TX (0.5%) and 2 × 10 min TBS. Lastly, the slides were coverslipped with Permount (Fisher Scientific). For each animal, three sections encompassing the intermediate grey matter were analyzed.

The number of TMR labeled cell bodies between the two spinal injuries was quantified. Tile scans of tissue sections were captured under epifluorescence using a 10x (0.3 NA) objective lens, and the number of TMR-positive cell bodies in the grey matter contralateral and ipsilateral to the T10 SCI was counted using the ImageJ cell counter plugin. To be considered a TMR-stained PrIN, each cell body had to meet three criteria: (1) contained TMR in the cytoplasm, (2) the nucleus was clearly visible based on its round shape and lack of TMR staining, (3) and at least one TMR-stained proximal process was clearly visible. Cell body counts were normalized to the rostrocaudal length (in *μ*m) of the section analyzed. To determine the ratio of soma between sides, we divided the average number of cell bodies/*μ*m on the side contralateral to the T10 SCI by the number of cell bodies/*μ*m on the side ipsilateral to the T10 SCI.

Tile scans were also used to count RtST collaterals and measure collateral growth. The numbers of collaterals branching from the RtST ipsilateral to the traced Gi (i.e., ipsilateral to the T10 SCI) into the grey matter were counted with epifluorescence using a 10x (0.3 NA) objective lens. Collateral numbers are reported as the average number of collaterals normalized to the average length (in *μ*m) of the analyzed sections and the total number of traced RtST axons. To assess collateral growth, RtST collaterals were manually traced on ImageJ. One section per animal was traced and a macro was created for ImageJ to automatically count the number of collateral intersections at 0–1000 *μ*m from the grey/white matter interface on the side ipsilateral to the traced side of the Gi. The ipsilateral side was used as the starting point, as only one side of the RtST was labeled with BDA and the majority of RtST axons descend ipsilaterally [28]. Collateral intersections were counted every 25 *μ*m and were normalized to the length (*μ*m) of the section analyzed and the number of traced axons.

For the analysis of reticulo-propriospinal contacts, z-stack image sets containing brightly stained TMR stained PrINs were acquired using a Leica DM4000 B confocal microscope equipped with a 40x (1.15 NA) objective lens. Identification of PrINs for analysis was based on the same criteria used to count PrIN cell bodies. Namely, cells identified as PrINs (1) contained TMR in the cytoplasm, (2) had a clearly visible nucleus with a round shape and lack of TMR staining (3), and had at least one clearly visible TMR-stained proximal process. Similar to the previous studies [29–33], contacts were identified based on three criteria: (1) the RtST bouton must be a round or elliptical swelling with a diameter twice that of the parent collateral; (2) there was no discernible gap between the RtST bouton and TMR-labeled dendrite/soma; and (3) both the RtST bouton and TMR-labeled dendrite/soma were in the same focal plane at the site of the putative contact. Appositions meeting these criteria have been shown to correspond to synaptic contacts (~90% correlation) at the electron microscopic level [34–39]. Additionally, to further validate these inclusion criteria for RtST bouton identification, boutons from all animals were examined for the presence of the synaptic marker synaptophysin in the same plane of focus (same z-plane) of confocal z-stacks (z-step size 0.6 *μ*m). Penetrance of the synaptophysin antibody used for immunofluorescence staining was low and so analysis was restricted to surface boutons on the top ~7–10 *μ*m of tissue sections. A total of *n* = 199 boutons were analyzed from *n* = 6 dSTAG animals, and *n* = 171 boutons were analyzed from *n* = 6 cSTAG animals. There were a few animals *n* = 2 dSTAG and *n* = 1 cSTAG where no surface boutons were found. In addition, we searched for RtST-PrIN contacts in which the contact was within 7–10 *μ*m of the surface (i.e., within the zone of synaptophysin labeling). Using this approach, we identified *n* = 12 RtST-PrIN surface contacts from *n* = 3 dSTAG animals. These contacts were examined for synaptophysin positivity in the same focal plane (same z) of confocal z-stacks (z-step size 0.76 *μ*m).

Identification of contacts was performed by scrolling up and down the z-stacks and finding appositions in the same plane of focus or ±1-step (0.76 *μ*m each). The average number of contacts in the grey matter contralateral and ipsilateral to the T10 SCI and the average total number of contacts were analyzed.

### 2.6. Statistical Analysis

Statistical analysis was carried out using SigmaPlot 13 (Systat Software Inc., San Jose, CA, USA) and GraphPad Prism 7 (GraphPad Software Inc., La Jolla, CA, USA). Normality was assessed using D'Agostino-Pearson omnibus test. Given a small *n*, the cSTAG group did not meet the conditions for normality. So, all between-group comparisons were made with Mann–Whitney *U* tests. The number of collateral intersections was assessed with two-way repeated measures analysis of variance (two-way RM ANOVA) and Tukey's multiple comparison test. Weekly BBB open-field locomotion scores were tested with two-way RM ANOVA and the Holm-Šídák method for post hoc analysis. Data are presented as mean values ± SEM, and the threshold for significance was set at a *p* value of ≤0.05.

## 3. Results

### 3.1. Behavioural Testing

**Figure 2:**
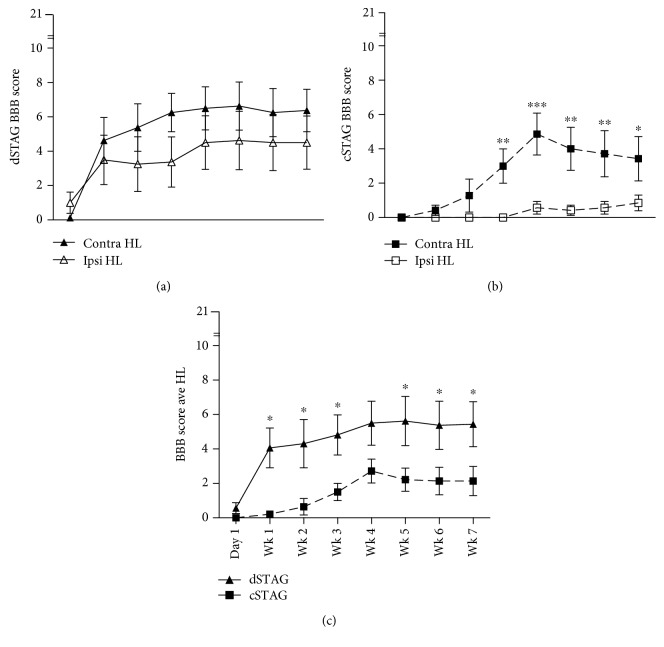
dSTAG animals displayed superior locomotor ability compared to cSTAG animals. Contralateral (contra) and ipsilateral (ipsi) hindlimb BBB scores of dSTAG rats were not significantly different (a), whereas contralateral hindlimb scores of cSTAG rats were significantly higher than ipsilateral hindlimb scores (^∗^*p* < 0.05) as assessed by 2-way repeated measures ANOVA. Post hoc testing showed that the contralateral hindlimb outperformed the ipsilateral hindlimb of cSTAG animals at weeks 3 (^∗∗^*p* < 0.01), 4 (^∗∗∗^*p* < 0.001), 5 (^∗∗^*p* < 0.01), 6 (^∗∗^*p* < 0.01), and 7 (^∗^*p* < 0.05). Average BBB score of the two hindlimbs was significantly greater for the dSTAG group compared to the cSTAG group (^∗^*p* < 0.05) from weeks 1 to 3 and 5 to 7 (^∗^*p* < 0.05; (c)). Error bars represent the SEM. Contralateral and ipsilateral are relative to the T10 SCI.

Overground open-field locomotion was evaluated using the BBB scale once every week for 7 weeks, beginning 1 day after T7 over-hemisection SCI. For dSTAG animals, contralateral and ipsilateral (relative to T10 SCI) hindlimb scores improved rapidly in the first week and then more slowly until about the third week postinjury for the contralateral hindlimb and the fourth week postinjury for the ipsilateral hindlimb (Figure 2(a)). Except for day 1 postinjury, the contralateral hindlimb of dSTAG animals showed consistently better motor function than the ipsilateral hindlimb, but these differences were not significantly different (Figure 2(a); *p* = 0.13). Conversely for cSTAG animals, contralateral hindlimb scores improved slowly over the first four weeks' postinjury, peaked at 4 weeks, and showed a steady decline in performance thereafter (Figure 2(b)). Motor function was almost absent in the ipsilateral hindlimbs of cSTAG animals until 4 weeks' postinjury and showed very slight improvement from weeks 4 to 7. Similar to the dSTAG animals, the contralateral hindlimb of cSTAG animals showed consistently better motor function than the ipsilateral hindlimb, with a significant main effect between the contralateral and ipsilateral hindlimbs (*p* < 0.05). The ipsilateral hindlimb of cSTAG animals had significantly lower BBB scores than the contralateral hindlimb at weeks 3 (^∗∗^*p* < 0.01), 4 (^∗∗∗^*p* < 0.001), 5 (^∗∗^*p* < 0.01), 6 (^∗∗^*p* < 0.01), and 7 (^∗^*p* < 0.05; Figure 2(b)). Taken together, there were clear differences in the progression of recovery of the contralateral and ipsilateral hindlimbs of the dSTAG and cSTAG groups.

Next, we examined the overall hindlimb motor performance of the dSTAG and cSTAG groups. Combined hindlimb BBB scores of both the dSTAG and cSTAG groups showed similar recovery progressions as the leg-specific BBB analysis (Figures 2(a), 2(b), and 2(c)). Comparison between the recovery progressions of the dSTAG and cSTAG groups showed that dSTAG animals recover motor function more rapidly (in the first week postinjury) and maintained a higher level of performance throughout testing. Statistical analysis of the performances over time showed that dSTAG rats had higher BBB scores than cSTAG rats at all time points except day 1 and week 4 (^∗^*p* < 0.05; Figure 2(c)). Collectively, these data indicate that dSTAG animals have a more consistent recovery of motor function between contralateral and ipsilateral hind legs, show a more rapid motor recovery after injury, and recover more hindlimb function than cSTAG animals.

### 3.2. Histology

#### 3.2.1. Lesion Size Was Assessed to Evaluate the Completeness of the Staggered SCI

**Figure 3:**
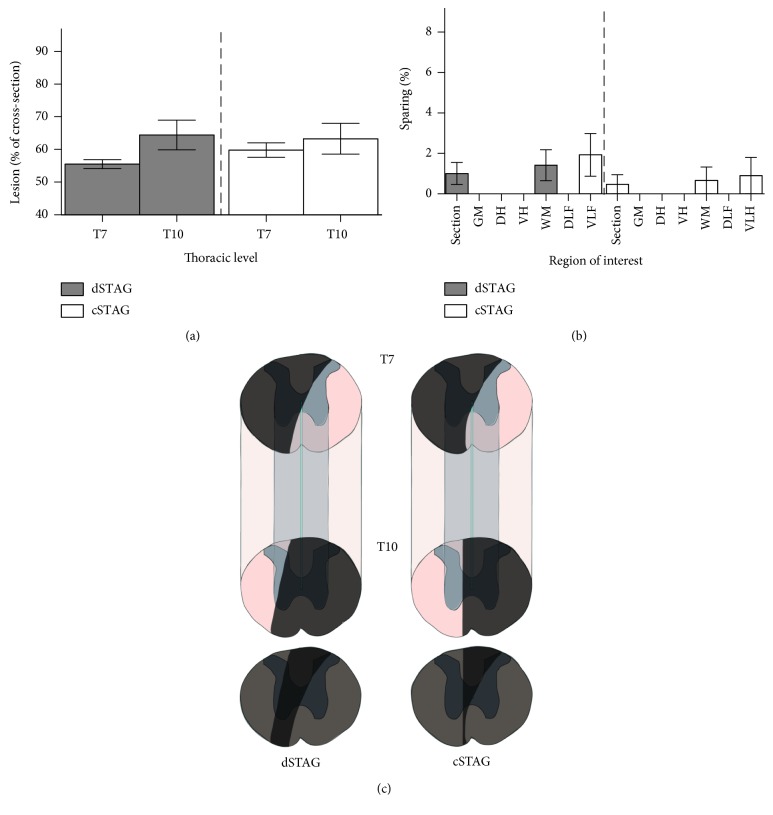
STAG SCI severity was unaffected by the inclusion of a time delay between injuries. The sizes of the T7 and T10 (a) spinal lesions as a percent of the cross-sectional area were comparable between groups. The majority of animals did not have spared tissue in different regions of interest when the T7 and T10 lesions were overlayed (a). The whole cross-section (section), grey matter (GM), dorsal horn (DH), ventral horn (VH), white matter (WM), dorsolateral funiculus (DLF), and ventrolateral funiculus (VLF) were analyzed for sparing (b). Schematics of spinal cord segments encompassing the lesions from representative dSTAG and cSTAG animals (c). Lesioned areas are shaded dark grey. White matter is seen ventrally at the level of the T7 lesion, but the T10 injury encroaches on the contralateral hemicord in both animals. Overlays of the T7 and T10 injuries are shown below the block schematics. When the two injuries are overlapped, the completeness of the lesion becomes evident. Error bars represent the SEM.

No significant differences in the cross-sectional areas of the T10 SCIs were found between the dSTAG (average 64.4% ± 4.5) and cSTAG (average 63.3% ± 4.7) groups (*p* = 0.87; Figure 3(a)). Similarly, no significant differences in the cross-sectional areas of the T7 SCIs were found between the dSTAG (average 55.6% ± 1.4) and cSTAG (average 59.8 ± 2.2; *p* = 0.07; Figure 3(a)). Although the goal was to transect only one half of the spinal cord at the T10 level, most injuries crossed the midline, thus affecting both sides of the spinal cord. Since the goal of our study was to determine the capacity of injured RtST axons to connect onto PrINs after SCI, ensuring transection of all RtST axons was preferred over taking the risk of injuries that were too small. Furthermore, although most T10 injuries crossed the midline, this did not prohibit the analysis of RtST-PrIN plasticity in the spinal tissue between the two SCIs because RtST fibers project in the lateral funiculus of the white matter [19].

Analysis of cresyl violet-stained sections revealed that there was no sparing in the grey matter, dorsal horns, ventral horns, and dorsolateral funiculi of the targeted side of the spinal cord. However, a small percentage (<4.2% of the total cross-sectional area; <7.9% of the ventrolateral funiculus area) of spared tissue was found near the RtST tracts in the ventrolateral funiculi at the T7 lesion of some animals (*n* = 4 dSTAG; *n* = 1 cSTAG; Figure 3(b)). To ensure that no animals had spared traced RtST axons, cross-sections caudal to the two SCIs were inspected for the presence of BDA-traced RtST axons. No traced axons were present caudal to the lesions in all cSTAG animals and most dSTAG animals (8 out of 9), indicating completeness of the SCIs. One dSTAG animal had 9.5% of traced RtST axons spared and was removed from the study.

#### 3.2.2. Counting of Traced RtST Axons and PrINs and Evaluation of Neuronal Tracer Injections

The accuracy of the bilateral tracer injections into the lumbar spinal cord to label PrINs was verified (Figure 1(b)). All animals had TMR tracer on both sides of the cord in the intermediate lamina, indicating success of PrIN tracing. The accuracy of tracer injections into the right Gi reticular nucleus to label the RtST was also verified (Figure 1(b)). All animals had a positive signal for neuronal tracer in the Gi reticular nucleus. Importantly, spread of the tracer to the contralateral side or pyramidal tract tracing (ventral to supposed injection site) was not found in any animal. Brainstem injection sites were in the Gi reticular nucleus of the majority of animals (*n* = 5 dSTAG; *n* = 5 cSTAG). The tracer injection site of the remaining animals (*n* = 3 dSTAG; *n* = 2 cSTAG) was in the intermediate reticular nucleus (Irt) with spread into the Gi. Nuclei adjacent to the tracing needle path showed some staining, including the prepositus nucleus (Pr), medial vestibular nucleus (MVe), external cuneate nucleus (ECu), solitary nucleus (Sol), and parvocellular reticular nucleus (PcRt). Given that none of these brainstem nuclei project to the thoracic spinal cord [40–44], we can conclude that tracer-positive fibers assessed in our study were likely of Gi origin.

**Figure 4:**
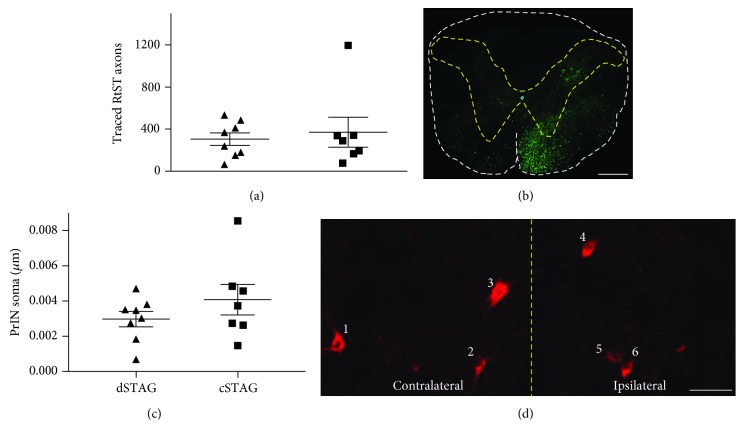
RtST-traced axon and PrIN counts. No significant difference in the number of traced axons was found between groups (a). Representative C1 cross-section showing the BDA-traced RtST (green; (b)). White dashed line, outline of cross-section. Yellow dashed line, outline of grey matter. Scale bar = 500 *μ*m (b). No significant differences in the number of soma in the grey matter were found between dSTAG and cSTAG groups (c). Six PrIN cell bodies in the bilateral grey matter (d). Yellow dashed line, midline. Scale bar = 50 *μ*m (d). Error bars represent the SEM. Contralateral and ipsilateral are relative to the T10 SCI.

No significant differences in the number of traced RtST axons were found between dSTAG and cSTAG animals (*p* = 0.87). The dSTAG group averaged 304 ± 60 traced axons, while the cSTAG group averaged 372 ± 142 traced axons (Figures 4(a) and 4(b)). PrIN cell bodies that were retrogradely stained with TMR and located in the intermediate grey matter between the T7 and T10 SCIs were counted from the contralateral and ipsilateral sides of the spinal cord relative to the T10 SCI and normalized to the length of the section analyzed. No significant differences between groups were found for the sum of TMR-positive soma on both sides of the grey matter (cell bodies/*μ*m; *p* = 0.54; Figures 4(c) and 4(d)). dSTAG animals averaged 29.8 × 10^−4^ ± 4.4 × 10^−4^ cell bodies/*μ*m, and cSTAG animals averaged 40.8 × 10^−4^ ± 8.7 × 10^−4^ cell bodies/*μ*m. There was a trend for higher numbers of TMR-positive soma in the contralateral hemicord compared to the ipsilateral hemicord of cSTAG animals. dSTAG animals averaged 15.1 × 10^−4^ ± 2.3 × 10^−4^ cell bodies/*μ*m on the contralateral side and 14.8 × 10^−4^ ± 2.6 × 10^−4^ cell bodies/*μ*m on the ipsilateral side. cSTAG animals averaged 25.1 × 10^−4^ ± 5.2 × 10^−4^ cell bodies/*μ*m on the contralateral side and 5.7 × 10^−4^ ± 4.1 × 10^−4^ cell bodies/*μ*m on the ipsilateral side. However, the contralateral/ipsilateral ratios did not statistically differ between groups (*p* = 0.07). Collectively, these data show that RtST and PrIN tracing was comparable between dSTAG and cSTAG.

#### 3.2.3. Counting of RtST Collaterals, Quantifying Collateral Growth, Counting Contacts between Collaterals and PrINs, and Assessment of Synaptophysin Signal

**Figure 5:**
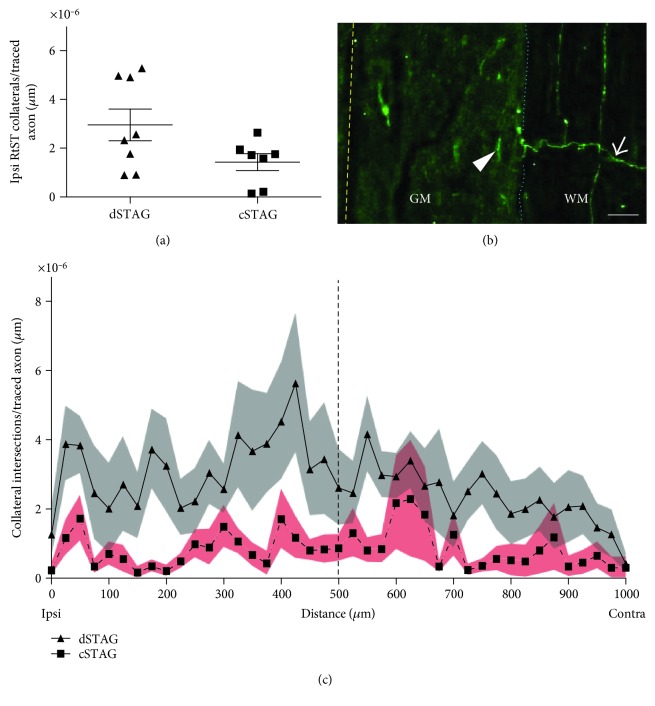
Traced RtST collateral counts and intersections at different distances from the grey/white matter border. Counts of traced RtST collaterals crossing into the grey matter did not differ significantly between groups (a). An ipsilateral (ipsi) RtST collateral (arrow) is seen projecting across the grey/white matter (GM/WM) interface (dotted blue line). Dashed yellow line, midline. Scale = 50 *μ*m (b). Line plot showing a greater number collaterals within the grey matter starting from the lateral grey/white matter border in the ipsilateral spinal cord (0 *μ*m) and continuing to the lateral edge of the contralateral (contra) grey/white border in the contralateral spinal cord (1000 *μ*m) for the dSTAG group. Dashed line, midline. Grey and red areas represent the SEM (c). Contralateral and ipsilateral are relative to the T10 SCI.

The average number of traced RtST collaterals that entered the grey matter (normalized to traced RtST axon count and the length of the section analyzed) was similar between dSTAG (3.0 × 10^−6^ ± 0.7 × 10^−6^ collaterals/traced axon/*μ*m) and cSTAG (1.4 × 10^−6^ ± 0.4 × 10^−6^ collaterals/traced axon/*μ*m) animals (*p* = 0.12; Figures 5(a) and 5(b)).

**Figure 6:**
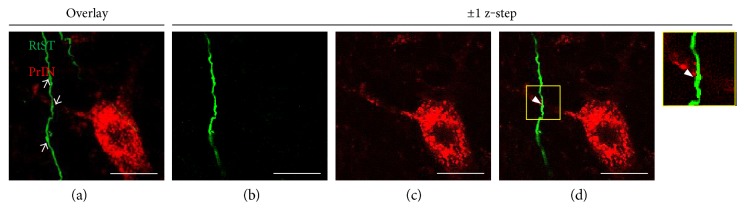
Close appositions between RtST boutons and PrIN neurons were considered contacts. Data from a dSTAG animal. RtST (green) and PrIN (red) channels (a–d). The arrows point to BDA-stained RtST boutons on a z-stacked image (a). RtST collateral (b) and PrIN soma (c) ± 1 z-step of each other. Combining the channels reveals a contact between a RtST bouton and PrIN dendrite (d). 1 z-step = 0.76 *μ*m. Scale = 20 *μ*m.

**Figure 7:**
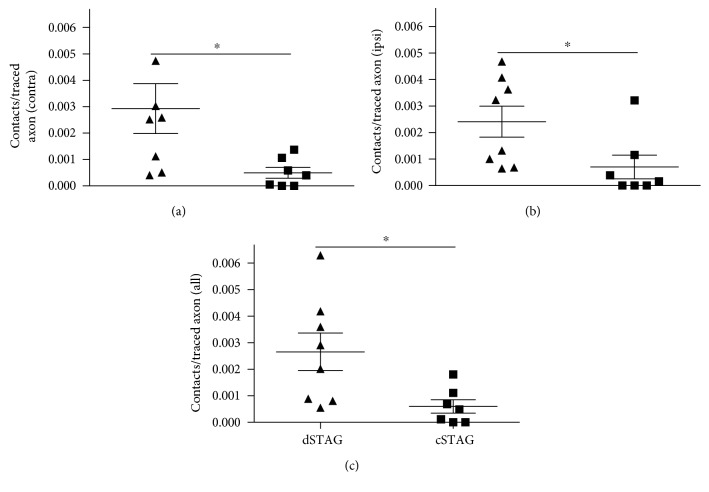
Higher numbers of reticulo-propriospinal contacts are found in dSTAG compared to cSTAG animals. dSTAG rats had significantly more reticulo-propriospinal contacts in the contralateral (contra (a); ^∗^*p* < 0.05), ipsilateral (ipsi (b); ^∗^*p* < 0.05), and bilateral grey matter of the spinal cord ((c); ^∗^*p* < 0.05). Contact numbers were normalized to the total number of traced RtST axons in all cases. Contralateral and ipsilateral are relative to the T10 SCI.

**Figure 8:**
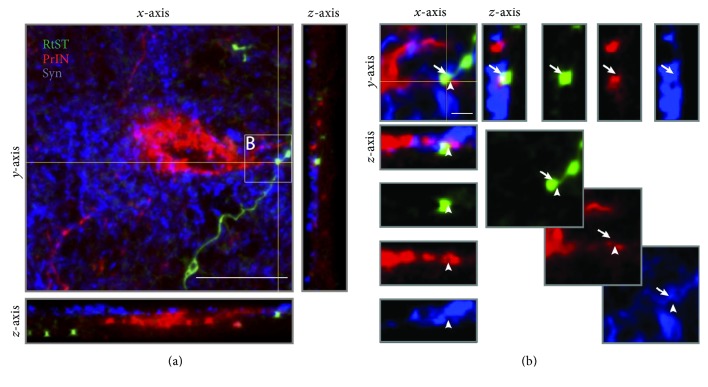
RtST boutons that form reticulo-propriospinal contacts contain synaptophysin (a). RtST collateral (green), PrIN cell body (red), and synaptophysin (blue). An orthogonal z-view of the *x*-axis is shown on the right hand side, and an orthogonal z-view of the *y*-axis is shown in the bottom. The thin yellow lines indicate the location of the orthogonal views. (b) High-magnification image of RtST bouton, forming a contact with PrIN cell body process. Individual channels are displayed diagonally, down, and to the right. Orthogonal z-views of the *y*-axis are shown on the right side and orthogonal z-views of the *x*-axis are shown in the bottom. Orthogonal views demonstrate colabeling of the bouton, process, and synaptophysin on the same focal plane. The site of contact is indicated by the arrowhead on the *x*-axis, and synaptophysin immunoreactivity within the RtST bouton is indicated by the arrow on the *y*-axis. Scale bar = 20 *μ*m (a). Scale bar = 5 *μ*m (b).

A significant main group effect was found on the number of collateral intersections 0–1000 *μ*m from the grey/white matter interface (normalized to traced axon count and length of section; *p* < 0.05^∗^; Figure 5(c)). Multiple comparisons showed that the dSTAG group had significantly more collateral intersections 425 *μ*m from the grey/white matter interface (*p* < 0.05). At this distance, dSTAG animals had 5.6 × 10^−6^ ± 2.0 × 10^−6^ collaterals/traced axon/*μ*m and cSTAG animals had 1.2 × 10^−6^ ± 0.6 × 10^−6^ collaterals/traced axon/*μ*m. Taken together, these analyses show that dSTAG animals had more extensive collateralization in the grey matter between injury sites than cSTAG animals. Contacts between RtST collaterals and PrINs were found in both ipsilateral and contralateral sides of the grey matter relative to the T10 SCI. An average of 18.0 ± 2.0 (dSTAG) and 14.7 ± 0.3 (cSTAG) TMR-stained PrINs per animal were analyzed on the contralateral side of the grey matter, and an average of 16.6 ± 1.6 (dSTAG) and 15.0 (cSTAG) cell bodies/animal was analyzed on the ipsilateral side.  Figure 6 shows an example of a contact from the dSTAG group. Contacts are reported as the number of contacts/traced RtST axon. In dSTAG animals, the number of reticulo-propriospinal contacts was higher in the contralateral side of the grey matter (^∗^*p* < 0.05; Figure 7(a)), the ipsilateral side (^∗^*p* < 0.05, Figure 7(b)), and the total of both sides (^∗^*p* < 0.05; Figure 7(c)) compared to that of cSTAG animals. The differences in connections were most pronounced in the contralateral spinal cord where the dSTAG animals had ~5.9 times more contacts than cSTAG animals (dSTAG: 29.3 × 10^−4^ ± 9.5 × 10^−4^; cSTAG: 5.0 × 10^−4^ ± 2.1 × 10^−4^), compared to the ipsilateral spinal cord where dSTAG animals had ~3.4 times more contacts than cSTAG (dSTAG: 24.1 × 10^−4^ ± 5.9 × 10^−4^; cSTAG: 7.0 × 10^−4^ ± 4.5 × 10^−4^). The dSTAG group also had ~4.4 times more total contacts than the cSTAG group (dSTAG: 26.6 × 10^−4^ ± 7.1 × 10^−4^; cSTAG: 6.0 × 10^−4^ ± 2.5 × 10^−4^). RtST boutons were further evaluated for synaptophysin immunoreactivity. Nearly all of the boutons examined from the dSTAG group (197 boutons out of 199) and nearly all of the boutons examined from the cSTAG group (169 boutons out of 171) were synaptophysin-positive, making it highly likely that RtST bouton-PrIN soma appositions were true synaptic contacts. Twelve RtST-PrIN contacts were also examined for synaptophysin immunoreactivity, and all were synaptophysin-positive, providing further evidence that contacts were functional synapses (Figure 8). To conclude, more contacts were found between RtST boutons and PrINs in dSTAG rats as compared to cSTAG rats.

## 4. Discussion

In this study, we show that transection of all traced RtST axons using a staggered spinal lesion model allows for rewiring of RtST axons onto PrINs that project to the lumbar spinal cord. Notably, this RtST plasticity occurs in parallel to improved locomotor performance. Considering the role of RtST axons in triggering locomotor activity [19, 45], these data suggest that rewiring of lesioned RtST axons might be a mechanism in locomotor recovery in our staggered SCI model. Interestingly, more synaptic connections with PrINs were found when the second SCI was delayed by 2 weeks (dSTAG) compared to animals that received both SCIs at the same time (cSTAG), which could explain the superior recovery of these animals.

Recent studies indicate that injured RtST axons can sprout and connect onto PrINs rostral to cervical lateral hemisections [9]. It was proposed that these connections were involved in promoting recovery, without taking into account that spared axons also increase their input into lumbar CPGs [5]. Here, we confirm that injured RtST axons can form contacts with PrINs after thoracic SCI with a staggered lesion model, where none of the traced RtST axons are spared. It is, however, possible that given the limitations of anterograde tracing techniques, not all RtST axons were labeled, and, so, there could have been spared untraced RtST axons in our staggered injury model. Nonetheless, the group of animals with the most RtST-PrIN connectivity showed the greatest improvements in locomotor function. Our data, however, do not consider the number of naturally occurring RtST collaterals projecting towards PrINs in intact animals, as this relation has already been established by Filli et al. [9].

The effect of temporally spacing out the two SCIs on RtST-PrIN contact formation cannot be accounted for by differences in lesion size or the number of traced PrIN soma and RtST axons, given that these measures were comparable between groups. The number of RtST axons entering the grey matter also did not differ between groups. However, dSTAG collaterals exhibited enhanced growth compared to cSTAG collaterals. Therefore, the rise in contacts in the dSTAG group compared to the cSTAG group might reflect an increase in total collateral length, rather than an increase in synapses per unit length of collateral. Alternatively, the group effect on contact formation might be due to a lack of excitability of PrINs in cSTAG rats. Following the more severe lesion scenario of the cSTAG group, spinal shock would have involved a greater spinal area and likely a longer time frame to resolve [46]; thus, making PrINs poor targets for the formation of new synapses in the first days following the lesion. Given that there is a window for heightened plasticity in the spinal cord in the weeks immediately following CNS damage [47], unresponsive PrINs in cSTAG animals during this early time window could have led to reduced RtST-PrIN contact formation. It is also possible that RtST-PrIN contact formation occurred during the time period between the resolution of spinal shock and time of neuronal tracing. To determine if this is the case, RtST-PrIN contact formation could be assessed in rats with SCI immediately following the resolution of spinal shock and compared to contact numbers several weeks after the resolution of spinal shock.

Superior recovery of dSTAG animals could also be because rats with spared white matter in one hemicord recover locomotor movements quickly and are very active in their cages (also because the leg opposite the SCI is largely functional) [5]. It is known that activity in the cage after SCI acts as a rehabilitative training [48] and promotes plasticity like the formation and consolidation of new contacts. Activity drives plasticity by inducing increases in growth factors BDNF and NT-3 in the spinal cord [49]. Furthermore, training after an incomplete SCI results in adaptations of spinal CPG networks, contributing to recovery. These adaptations remain functionally relevant even after subsequent complete spinal transections in cats [50] and rats [51]. In fact, cat [52] and rat [53] spinal CPGs can be trained to a point that animals can recover weight support and stepping movements on a treadmill. Consequently, by the time dSTAG animals receive the second injury (2 weeks following the first SCI), the spinal cord has already adapted and, thus, these animals have a greater capacity for locomotion. Since cSTAG animals can hardly generate movements, self-training and the resulting plasticity are hindered. That is to say, activity below the lesion is likely a key for rewiring.

Our results showing some modest recovery of hindlimb motor function in cSTAG animals are consistent with other studies from our lab using the cSTAG model [12, 25] but in contrast with other studies [10, 54] where rodents that underwent cSTAG hemisections recovered very little. One possible explanation for this discrepancy is that the methods of locomotor assessment were different between studies, making it challenging to compare outcomes. For example, in van den Brand et al. [10], rats were placed in an artificial upright bipedal posture. In Courtine et al. [54], recovery was assessed with EMGs and kinematics while mice walked on a treadmill. On the other hand, in our study, the BBB locomotor rating scale was used for behavioural assessment. Alternatively, the discrepancy in functional recovery of cSTAG animals may be related to the completeness of the lesions in the present study. However, only 1 of 7 cSTAG animals had spared white matter in the ventrolateral funiculus (Section 3.2). Although we found an absence of traced RtST axons in the sublesional spinal cord, as little as 5% sparing in the ventrolateral white matter can support recovery of up to a score of 8 on the BBB scale (rhythmic movements of the two hindlimbs) [19]. Spared white matter in the cSTAG animal with sparing in the present study possibly contained uninjured fibers from descending tract systems of non-Gi origin, such as the vestibulospinal or rubrospinal tracts. Hence, small spared white matter areas could have influenced the behavioural outcome of one of the cSTAG rats in our study.

Functional recovery in the present study is consistent with Courtine et al. [54] in that animals with delayed contralateral hemisections recovered better than animals with simultaneous injuries. Courtine et al. [54] speculated that supraspinal-propriospinal relay connections were responsible for the observed recovery in rats with a delay between the hemisection injuries but did not provide evidence of these connections. In our experiment, we provide evidence of increased numbers of RtST-PrIN connections in animals with temporally separated lesions compared to simultaneous lesions. Furthermore, RtST-PrIN connections occurred in parallel to functional recovery. However, a causal relation between the connections and functional changes remains to be established. A possible approach to establish causality would be to reversibly shut down PrINs in the tissue bridge connecting staggered SCIs and assess the function. For example, PrINs could be double infected with a retrograde gene-transfer lentiviral vector in the lumbar spinal cord and an adeno-associated vector at the soma [55]. The lentiviral vector would transfer a tetanus neurotoxin gene downstream of the tetracycline-responsive element, while the adeno-associated vector would carry a Tet-ON sequence (a type of reverse tetracycline transactivator). Double-infected PrINs could, then, be selectively and temporarily shut down via doxycycline-dependent transcriptional activation of the tetanus neurotoxin gene [55]. Alternatively, PrINs could be reversibly silenced with designer receptors exclusively activated by designer drugs (DREADDs) technology [56]. A limitation of reversibly silencing PrINs is that resulting functional drops could be due to the shutting down of connections between descending axons and PrINs or shutting down of PrINs alone. Indeed, plasticity in CPG networks can support some degree of motor recovery post-SCI by itself. For example, Martinez et al. [50] showed that adaptations of motor function occur in cats with incomplete SCI, and some of these adaptations are retained after subsequent complete spinal cord transection. Further, Cowley et al. [57] induced locomotor-like activity by stimulating the brainstem electrically soon after cSTAG SCI in an *in vitro* preparation. In other words, plasticity of descending systems over-time post-injury was unnecessary for transmission of locomotor command signals to the spinal cord. To determine whether RtST descending input is needed for the support of motor recovery in the staggered SCI model *in vivo*, neurons of the RtST projecting to the tissue bridge between hemisection injuries could be reversibly silenced using double infection techniques [55] or DREADDs technology [56]. Also, silencing data could be complemented with electrical stimulation of brainstem nuclei giving rise to the RtST and electromyographic recordings of hindlimb muscles [58].

It is noteworthy that in the cSTAG group the hindlimbs ipsilateral to the T10 SCI were nearly paralyzed as compared to the contralateral hindlimbs. Contralateral hindlimb function of the dSTAG and cSTAG groups was similar. However, unlike cSTAG, the ipsilateral hindlimb of dSTAG animals recovered to the point where there was no difference between sides (Figures 2(a) and 2(b)). In other words, locomotor benefits of temporally separating the two spinal lesions are attributable to improvements of the ipsilateral hindlimb. The differences in function between the contralateral and ipsilateral hindlimb are consistent with the location of the SCIs (Figure 1(c); the left lesion was at vertebral level T7 above the right lesion at level T10). Following the two injuries, RtST axons would have descended on the ipsilateral side and innervated lumbar projecting PrINs there. Most PrINs are single-crossing (crossing the midline once only) [7, 59]. Therefore, most of the innervated PrINs would have projected to the contralateral side, helping restore contralateral/left axis function (Figure 1(c)). It is also possible that descending RtST axons innervated double-crossing PrINs to restore the ipsilateral/right axis, but these double-crossings are relatively rare [9]. Although the RtST descended on the right, it sprouted to the opposite side, contacting PrINs there. These PrINs could have improved the contralateral/left axis via unilateral projections or the ipsilateral/right axis via single-crossings (Figure 1(c)). On the whole, fewer routes of communication were possible for the ipsilateral leg. Increased RtST-PrIN contact formation in dSTAG animals could have provided the missing input to the ipsilateral leg to support recovery.

Though our findings of RtST-PrIN connections strongly support the idea that they contributed to recovery, it is also possible, and even likely, that other pathways were involved in the recovery. Pontine reticular, vestibulospinal, and rubrospinal pathways have all been implicated in the modulation of locomotion [60, 61]. In addition to these pathways, the CST can form relays with PrINs in rodents with staggered hemisection SCIs [10]. The CST, moreover, has a causal effect on the locomotor function of staggered rats, as shown by the loss of locomotion following cortical inactivation with the GABA agonist muscimol [10]. Although convincing, these results are surprising as rodents without any CST input are able to walk effectively over open ground [62]. Taking into consideration the multiplicity of descending control of locomotor networks, our earlier work with the staggered SCI model on adaptations in motor neuron properties [12] and the current study's results, it becomes increasingly clear that even in experimental SCI, the contribution of plasticity at different levels to recovery is complex and not yet fully understood.

Neuroplasticity is a key mechanism in functional recovery after SCI in both animal models and humans. As reviewed by Raineteau and Schwab [63], several studies have indicated that the main functional adaptation following SCI is synaptic plasticity in pre-existing pathways and the formation of new circuits through collateral sprouting of injured and uninjured axons [6–10]. Therefore, continued investigation of the exact spinal tracts and systems involved and nature of the circuit rewiring is of the utmost importance to understand the full complexity of the mechanisms of recovery. Complete knowledge of the mechanisms of recovery can then be harnessed to discover and inform new treatments for individuals living with SCI.

## 5. Conclusions

Our work shows that injured RtST axons form synaptic contacts with lumbar-projecting PrINs after SCI in rats. Further, RtST-PrIN contact formation is paralleled by locomotor recovery, suggesting RtST rewiring is contributing to locomotor recovery post-SCI. Finally, a time delay between SCIs in the staggered injury model allows for immediate post-SCI activity, resulting in greater plasticity compared to simultaneous injuries.
